
JOURNAL INFORMATION
==============================
NLM Title Abbreviation: Atlanta Med Surg J (1884)
NLM Journal ID: 101251623

Atlanta Medical and Surgical Journal (1884)

Publisher: Biomedical Journal Digitization Project

ARTICLE INFORMATION
==============================
PMCID: PMC9201849
PMID: 35829604
Article version: 1
Subjects: Editorial

The National Quarantine Convention at Memphis

Print publication date: 1899 Jan
Volume: 15
Volume Series: New Ser
Issue: 11
First page: 745
Last page: 749
License: This work is free of known copyright restrictions. For more information, please see PMC Back Issue Digitization.
License URL: https://creativecommons.org/publicdomain/mark/1.0/

==============================

### *EDITORIAL NOTES AND COMMENTS.*

---

The Business office of THE JOURNAL is 305, 306 Fitten Building.

The Editorial office is Room 401, 402 Grand Opera House.

Address all Business communications to Dr. M. B. Hutchins, Mgr.

Make remittances payable to THE ATLANTA MEDICAL AND SURGICAL JOURNAL.

On matters pertaining to the Editorial and Original communications address Dr. Dunbar Roy, Grand Opera House, Atlanta.

Reprints of original articles will be furnished at cost price. Requests for the same should always be made *on the manuscript.*

We will present, post-paid, on request, to each contributor of an original article, twenty (20) marked copies of THE JOURNAL containing such article.

---

### THE NATIONAL QUARANTINE CONVENTION AT MEMPHIS.

THIS Convention of representatives from the South and Southwest recently held a very satisfactory meeting in Memphis to consider the question of quarantine.

The *Boston Medical and Surgical Journal* has written an editorial on the work accomplished by this meeting, and we could do no better than quote what it says:

"The Memphis Quarantine Convention, to which reference was made in the last issue of the *Journal*, ought to bring us within measurable distance of a settlement of the vexed question of the nature and limits of the quarantine service of the country.

"The call for the meeting, issued by the Memphis Chamber of Commerce, was addressed to those only who were in favor of a comprehensive control of quarantine under the authority of the National Government, and thus were cleared away many of the difficulties which have hitherto beset such conventions.

"Nearly one hundred and fifty delegates were present, representing States, cities, commercial organizations and some of the great railroad systems of the Southwest. Though many medical men were present as delegates from the various public health authorities of the country, still the physicians were, upon this occasion, in a minority. The South was, naturally, more fully represented than the North, and the State of Mississippi, which suffered more severely from the effects of yellow fever panic than did any other Southern State during the recent epidemic, had the largest delegation of all.

"The men composing the delegations are distinguished in many walks of life. The very efficient Chairman of the meeting was Gen. Luke E. Wright, of Memphis, formerly Attorney-General of his State. At the head of the Mississippi delegation was ex-Governor Shands, professor of law in the University of Mississippi.

"Ex-Governor Bullock, of Georgia, was an active and influential participant in the work of the body, both in committee-room and on the floor of the Convention, and was ably seconded by the very capable Mayor of Atlanta, Mr. Collier. The Chairman of the Louisiana representation was a very prominent member of the Cotton Exchange of New Orleans, and though he had been instructed by that organization to resist the attempts of 'the doctors' to obtain possession of the administration of quarantine, was able to support heartily and ably the resolutions which were finally adopted.

"Under the energetic direction of the Chairman of the Convention, no time was lost in the usual and useless discussions upon the nature and proper treatment of yellow fever, for he discovered the happy expedient of relegating several papers upon these subjects, together with an exhaustive statement of our duty to the new possessions of the United States beyond the seas, to the evening sessions of the Convention, while the sub-committees of the meeting were at work upon the matters referred to them.

"For a proper consideration of the difficulties which attend such Conventions as this, in Southern States, it should always be remembered that *yellow fever* is the one disease always present in the minds of the Southern people when the question of quarantine is under discussion, and it is quite apparent that this collection of men offered no exception to the rule. A considerable number of those present, especially among the representatives of the transportation companies, would undoubtedly have preferred to vote for some measure which gave absolute power to the general government in times of yellow fever epidemic and to stop there, rather than to go on with the consideration of a general system for the protection of the public health. It was well therefore for Dr. Hamilton, one of the delegates from Illinois, to call the attention, as he did, of the Convention to the necessity of a wider view of the great subject of the spread of preventable diseases other than yellow fever. Dr. Hamilton's complete familiarity with the work of the Marine Hospital Service gives to his opinions upon a national public health service great value—a value which has not been essentially diminished by the apparent uncertainty as to the disinterested quality of his utterances upon this subject.

"He represents the recorded opinions of the great medical associations of the land and of the most influential public health organizations, and they are all in favor of some national organization, intelligent enough to comprehend the great problems presented by preventable diseases, and strong enough to use all the power of the general government in case of need, to control the spread of pestilence.

"The Convention devoted two days to the consideration of the question of the best form of national organization, and as a result of their deliberations, adopted the resolutions which we have already printed.

"These resolutions were prepared by a committee consisting of one delegate from each State represented in the Convention; this Committee was presided over by ex-Governor Shands, of Mississippi, and was again not under the control of the medical profession, and was able to come to a unanimous report. The report was adopted by a vote of 136 to 4, and was then upon motion of a member of the minority made unanimous.

"Those of our readers who are familiar with the proceedings of a quarantine convention in a Southern city will understand how important a result has been reached by this united report.

"For the general interest of scientific medicine it is to be hoped that some agency may now be established for a wise and patient investigation of the one preventable exotic disease which is of more consequence than any other to this country. It is true that now and again officers of some of the medical services of the government at Washington have been sent into the tropics for brief periods of time to study yellow fever, but thus far we have gained little by such efforts, and cannot hope to do so until such studies can be carried on under the direction of a well-established department of government which shall not be compelled to fight for its existence at every session of Congress—as was the case with the National Board of Health.

"The conditions which can meet the requirements of the resolutions of the Memphis Convention are substantially those of the so-called Spooner Bill, now before Congress, for the creation of a Bureau of Public Health with a commissioner at its head aided by a council composed of a delegate from each State. The council would serve many useful purposes: It would unite the activities of the various States in health matters, it would bring about an immediate connection between the authorities of the States and the office at Washington; every member of the council, having had experience in public health administration in his own State, would be a useful counsellor in the new board; and finally would exercise a most important function, in procuring, each in his own State, that support on the floor of Congress which is essential to the satisfactory maintenance of a public department, a support which cannot easily be refused to one who can speak with authority among the constituents of the respective members of Congress."
